# Its Architecture, Planning, and Control

**Published:** 1912-04-27

**Authors:** 


### THE IDEAL SCHOOL CLINIC.

### Its Architecture, Planning, and Control.

During the last few years various educational authorities, both in this country and abroad, have established their own school clinics. The work of designing and planning these institutions, in cases where an entirely new clinic was desired, was in most cases left in the hands of the municipal architect—sometimes a gentleman whose acquaintance with hospital construction and the demands of an out-patient department were of the most rudimentary kind. In a few cases clinics have been adapted from already existing buildings, usually those occupied by the municipal health or sanitary staff; an example of the adapted style is the Abertillery clinic, of which we gave a description in a recent article. In any case there is at present nothing that can be taken as a type of the ideal clinic in existence. Doubtless in course of time as these institutions become more popular and the demand for them increases, architects will begin to study the matter seriously and consider what styles of construction are best suited for them. But at present every clinic is an attempt to attain to something which will help to realise the ideal of the future; an experimental design which very often is open to grave objections.

Regarded purely from a structural point of view, the ideal school clinic should be a miniature out-patient department, with this important difference: that it is an out-patient department which is highly specialised and in which concentration reaches its acme.

The main essentials of such a department are simple. They comprise: (1) Adequate accommodation for the patients, by which is understood waiting-room accommodation, together with lavatory accommodation; (2) rooms for the almoner or inquiry officer, clerk, or whoever may be in charge of the secretarial work of the clinic; (3) dispensary; (4) rooms for examining and treating patients; (5) a small operating room; (6) a bathroom; and lastly (7) accommodation for the nurses and staff in charge of the clinic. It may seem that these requirements, regarded as essentials, are greater than at present are recognised as indispensable in a school clinic. We answer to this objection that most modern clinics are makeshifts, and that what we are here concerned with is the ideal institution in which the maximum of efficiency is secured with the minimum of effort. Already architects are devoting considerable attention, in collaboration with medical experts, to the construction of modern schools (see Baginsky's authoritative work), and it is to be hoped that they will soon become as interested in the question of school clinics as well.

Let us take these requirements in some detail. First as to the general type of the clinic. It is highly desirable that the clinic should be a separate one-storeyed building, no matter whether it is attached to a school or whether it stands alone at some distance from its contributing centres. The reasons for this isolation are too self-evident to need demonstration. It is, of course, perfectly possible to do good work in an old-fashioned room which has been generously placed at the disposal of the medical staff by the school authorities, but such an arrangement must be regarded as merely temporary, and no stretch of imagination can picture the ideal clinic under such conditions. A simple brick building with tiled roof, with a double T-shaped ground plan, is the type favoured by most authorities. The usual hospital conditions should be secured; thus the building must be placed with its long axis pointing S.E. and N.W. so as to secure the maximum amount of sunshine. In all other respects the architect should bear in mind that the building is destined to serve as an out-patient department and not as a class-room, and that it is therefore desirable that provision should be made for securing cleanliness and aseptic environment on the same basis as in a hospital.

The central portion of the building is devoted to what may be called the administrative part of the clinic. A fairly large waiting-room, with two smaller side rooms, one for the almoner or inquiry officer and the other for the dispensary, must be provided. The waiting-room must possess adequate seating accommodation, preferably in the shape of removable benches; the floor must be of smooth hard wood or of linoleum, so as to serve as a "crawl ground" when the hall is used for orthopædic exercises on the Klapp plan. Separate entrances for patients coming and going are desirable, and lavatory accommodation must be provided for in rooms easily accessible from the central hall, yet sufficiently isolated to give the requisite privacy.

In the wings the examining and treatment rooms are located. Here, too, a sitting-room must be provided for the nurses, together with private lavatory accommodation. A small retiring-room for the members of the medical staff, with toilet and lavatory provision, should also be set apart. As hot water is indispensable, arrangements must be made to secure a constant supply. This is best secured by a basement boiler, although it may also be drawn from a small kitchen built in the rear of the waiting hall. No matter what style of plan is adopted, the design must be so drawn that the accommodation for the patients and for the staff of the clinic is sufficiently demarcated so as to ensure the necessary privacy to the latter.

Who is to be in charge of the clinic? This question is easily settled when the institution is built as an adjunct to a school; in that case the school caretaker, residing on the premises, may be entrusted with the task of looking after the clinic buildings at night, though it is always preferable that a separate caretaker for the clinic itself should be provided. Such a caretaker need not be residential, and there is therefore no need to provide him with rooms on the premises. His duties will be to see that the boiler is in proper working order and that the fires are kept up, the rooms cleaned, the refuse removed, and the clinic generally kept in order. Where a nurse is expected to reside on the premises adequate living accommodation must be provided for her; in that case the cost of the clinic will be much increased, without, it is to be feared, affording a corresponding advantage from an administrative point of view. For this reason it is better that the nurses in attendance should be non-residential like the members of the medical staff. This is the case in nearly all the school clinics which are at present in existence, whether voluntary or municipal.

(*To be continued.*)

